# Silicon Quantum Dot Light Emitting Diode at 620 nm

**DOI:** 10.3390/mi10050318

**Published:** 2019-05-11

**Authors:** Hiroyuki Yamada, Naoto Shirahata

**Affiliations:** 1 International Center for Materials Nanoarchitectonics (MANA), National Institute for Materials Science (NIMS), 1-1 Namiki, Tsukuba 305-0044, Japan; yamada.hiroyuki2@nims.go.jp; 2 Department of Physics, Chuo University, 1-13-27 Kasuga, Bunkyo, Tokyo 112-8551, Japan; 3 Graduate School of Chemical Sciences and Engineering, Hokkaido University, Sapporo 060-0814, Japan

**Keywords:** quantum dot, silicon nanocrystals, light emitting diode

## Abstract

Here we report a quantum dot light emitting diode (QLED), in which a layer of colloidal silicon quantum dots (SiQDs) works as the optically active component, exhibiting a strong electroluminescence (EL) spectrum peaking at 620 nm. We could not see any fluctuation of the EL spectral peak, even in air, when the operation voltage varied in the range from 4 to 5 V because of the possible advantage of the inverted device structure. The pale-orange EL spectrum was as narrow as 95 nm. Interestingly, the EL spectrum was narrower than the corresponding photoluminescence (PL) spectrum. The EL emission was strong enough to be seen by the naked eye. The currently obtained brightness (∼4200 cd/m^2^), the 0.033% external quantum efficiency (EQE), and a turn-on voltage as low as 2.8 V show a sufficiently high performance when compared to other orange-light-emitting Si-QLEDs in the literature. We also observed a parasitic emission from the neighboring compositional layer (i.e., the zinc oxide layer), and its intensity increased with the driving voltage of the device.

## 1. Introduction

Solid-state lighting in the form of light emitting diodes (LEDs) is expected to reduce global energy consumption in the lighting industry [1,2]. Unlike phosphor-coated chips that control the current commercialized LEDs, electric-driven LEDs offer advantageous properties including structurally admissible heavy carrier injection compared to phosphor-coated devices [3,4,5].

Devices with active layers of colloidal quantum dots (QDs) of semiconductors offer an advantageous electroluminescence (EL) performance, including color purity, high luminance (∼200,000 cd/m^2^), narrower spectra for emission (full-width at half maximum, fwhm < 40 nm), spectral tunability of the emissions over a broad wavelength range from ultraviolet to near-infrared through to full-color of visible, an operation voltage as low as 3 V, a stable emission under long-term operation even at high current-density conditions, and a solution-based processability [6,7,8]. The best values of external quantum efficiencies (EQEs) of red-emitting quantum dot light emitting diodes (QLEDs) with conventional and inverted structures are currently 20.5% and 18.0%, respectively [9,10]. These magnitudes are close to an energy conversion efficiency of a mercury lamp which works as a benchmark for the industry. The high values of EQE require optically active (or emission) layers of cadmium-based QDs such as CdSe covered with a shell of crystalline ZnS. However, Hazardous Substances (RoHS) strongly restrict the use of toxic elements, including Cd, for electronic products. Due to the complete ban of these elements in the future, recent efforts have shifted toward fabricating heavy-metal-free QLEDs. More recently, Yang and co-workers reported the InP/ZnSe/ZnS-based red QLED with a 6.6% EQE [11]. Xu and co-workers reported a pale-orange-emitting (λ_em_ = 625 nm) QLED in which colloidal QDs consisting of CuInS_2_-ZnS-alloyed (ZCIS) cores and ZnSe/ZnS double shells work as an active layer [12]. The 6.6% EQE is the current record of EQE for solution-processed QLEDs exhibiting visible emission.

Silicon (Si), which is abundant, is poised to become a safe alternative to Cd-based QDs [13]. Many studies have concluded that Si is nontoxic to the environment and the human body [14]. Bulk crystalline Si exhibits poor optical performance due to the indirect bandgap nature, but the confined carriers in the nanocrystal with a diameter smaller than the bulk exciton Bohr radius (~5 nm) induce a change in the energy structure. This situation allows for the overlapped wave functions of spatially confined carriers, leading to zero-phonon optical interband transitions for recombination, as a result of the relaxation of the k-selection rule due to the Heisenberg uncertainty relation [15]. Passivation of freestanding silicon quantum dots (SiQDs) with hydrogen atoms is the simplest way to form a surface that negligibly influences the optical properties. Hydrogen-capping gives nonpolarity to a surface, allowing the highest coverage while avoiding a surface that can remain unpassivated. Therefore, most studies on theoretical modeling use the hydrogenated surface to investigate the effect of quantum confinement (QC) generated in a “pure” SiQD. Analogous to the QDs of other semiconductors, the space-confinement-induced changes of the energy structure is expected to enhance the photoluminescence quantum yield (PLQY), but the values remain low (~5%) for ncSi:H [16].

Simply substituting the SiQDs’ surface hydrogen atoms with alkyl chains, which yields a covalent carbon–silicon linkage, their PLQYs increase to ~65% at maximum [17,18,19,20]. Such an enhancement has been arguably observed for alkyl-terminated SiQDs with size-dependent PL bands peaking in the 590–1130 nm range [16,21,22]. Currently, the enhancement is postulated to arise from an increase in the radiative recombination rate [19], a dramatic reduction of the nonradiative channels [20,23], or a bandgap modulation from indirect to direct transitions [24]. Such high values of the PLQY are suitable as active layers for the QLED. To date, the EL spectra over a wavelength range from 625 to 850 nm have been reported from Si-QLED devices. The record values of EQE are as high as 8.6% for near-infrared EL [25], 6.2% for red EL [26], 0.03% for orange EL [27], and 0.03% for white EL emissions [28]. The shortest emission wavelength is currently around 625 nm for a Si-QLED, but its EQE is as low as 0.0006% [27]. In order to form an image of superior color rendering, the enhanced EQE of a pale-orange-light emitter (i.e., 600–630 nm range) is a challenging task.

In this study, we synthesized the colloidal ink of a pale-orange fluorescent SiQD with an 8% PLQY. The QD was used for the preparation of a Si-QLED with an inverted device structure. The Si-QLED exhibits the EL spectrum peaking at 620 nm, which is included in the pale-orange emission wavelength range.

## 2. Materials and Methods

### 2.1. Reagents and Materials

Triethoxysilane (TES) was purchased from TCI chemicals (Tokyo, Japan). 1-Decene was purchased from Sigma-Aldrich (Saint Louis, MO, USA) and was used as received. Electronic grade hydrofluoric acid (49% aqueous solution, Kanto Chemical, Tokyo, Japan), Toluene (High Performance Liquid Chromatography, HPLC, grade), dicholrobenzene, ethanol, and methanol were purchased from Wako chemical (Tokyo, Japan). Colloidal ink of zinc oxide (ZnO, Sigma-Aldrich), 4,4′-bis(carbazole-9-yl)biphenyl (CBP, 99.9% trace metals basis, Sigma-Aldrich), and molybdenum (VI) oxide (MoO_3_, 99.97% trace metals basis, Sigma-Aldrich) were used as received. Water was purified and deionized using a Sartorius (Arium 611 UV, Sartorius AG, Göttingen, Germany) water purification system.

### 2.2. Preparation of Silicon Quantum Dots (SiQDs)

The synthesis of SiQDs was performed in a two-step process, according to our previous papers [28,29]. Typically, TES was employed as a starting precursor. The hydrolysis product, i.e., (HSiO_1.5_)_n_, of the TES was thermally disproportionated at 1050 °C for 2 h in 5%/95% H_2_/Ar atmosphere, yielding SiQDs dispersed in a SiO_2_ matrix. After cooling to room temperature, 300 mg of the brown solid (i.e., Si/SiO_2_ composite) as powder was mechanically ground in an agate mortar with a pestle. The fine powder obtained was stirred for ~1 h in a mixture of ethanol and 48% HF (aq) to liberate the QDs from the oxide. Then, the acidic solution was centrifuged at 15,000 rpm and washed with ethanol and acetonitrile in that order. According to the analysis with Fourier transform infrared (FTIR) spectroscopy, the precipitated product was a SiQD terminated with hydrogen atoms. Thermal hydrosilylation was carried out in 1-decene at 200 °C. We obtained a transparent brown-colored solution at the same time as the solution temperature reached 200 °C. The unreacted 1-decenes were removed by a vacuum evaporator. Finally, the chloroform solution of the product was purified and separated by gel permeation chromatography (GPC). The substitution of hydrogen atoms by a 1-decane monolayer, yielding a carbon–silicon covalent linkage (i.e., decane-terminated SiQD, SiQD-De), was experimentally confirmed by FTIR. The GPC-treated samples were dried under vacuum conditions and stored in Ar atmosphere prior to use for device fabrication.

### 2.3. Device Fabrication

Devices were fabricated on a glass substrate. A 150 nm thin film of indium tin oxide (ITO) uniformly sputtered on the glass gives a resistivity of 10–14 Ω/sq, which is good value for EL device fabrication. ITO-coated substrates were prepared in a manner similar to conventional device fabrication. Next, the colloidal ink of ZnO was spin-coated with a rotation speed of 2000 rpm. After baking the film at 120 °C in air, the emission layer of the SiQD-De was spin-coated with a concentration of 10 mg/mL in toluene with a speed of 1500 rpm. Then an organic layer of CBP with a thickness of 40 nm was thermally evaporated. A 30 nm MoO_3_ layer was deposited with a vacuum level of 10^−5^ Pa by thermal evaporation. Then the Al top electrode with a thickness of 150 nm was deposited to mask over the film.

### 2.4. Optical Properties

Optical absorbance spectra were recorded using an ultraviolet-to-visible (UV–VIS) spectrophotometer (JASCO V-650, Tokyo, Japan) with an integrated sphere by diffuse reflection setup. A PL measurement at room temperature was carried out with a spectrofluorometer (NanoLog, Horiba Jovin Yvon, Tokyo, Japan). For measuring the PL and PL excitation (PLE) spectra, we prepared a chloroform solution of SiQD-De. Absolute PLQYs were measured by the standardized integrating sphere method (C9920-02, Hamamatsu Photonics, Hamamatsu, Japan). To avoid the solvent effect on the PLQY, the values of the PLQYs were measured using the SiQD-De films deposited onto quartz glass substrates. The peak value of the PLQY was estimated to be ~8%. This value is lower than those of red-emitting SiQDs and near-infrared (NIR)-emitting SiQDs but is close to the values for the reported SiQDs which exhibit PL spectra peaking in the pale-orange color range.

### 2.5. Calculation of External Quantum Efficiency (EQE)

EQE was calculated as the ratio, per unit time, of the number of forward-emitted photons to the number of injected electrons, *I*_d_/|*e*|, where *I*_d_ is the current passing through the QLED device at an applied bias, V. We can express this as

(1) $$\mathrm{EQE}(\%)=N_{\mathrm{phot}}\times |e|I_{d}\times g\times 100$$

where *N*_phot_ is the number of forward-emitted photons actually collected by the photodiode and the geometric factor, *g*, accounts for the solid angle of the EL profile (assumed to be Lambertian) subtended by the photodiode, Ω = π⁄g:

(2) $$g=\;(a^{2}+L^{2})/a^{2}$$

where *a* is the diameter of the active area of the photodiode and *L* is the distance between the emitting QLED pixel and the photodiode. *N*_phot_ was calculated from the photocurrent output of the photodiode in response to the detected EL. The photodiode current, divided by the responsivity value of the photodiode at the peak wavelength of the EL curve, gives the light output power from the LED device. Then the number of photons is calculated by just dividing with *hc*/*λ*. Therefore, the simplified formula we used for EQE is

(3) $$\mathrm{EQE}(\%)=\frac{I(\mathrm{Photodiode})\times \lambda (\mathrm{peak})\times \mathrm{electron}\;\mathrm{charge}\;\times g\times 100}{R(\lambda )\times hc\times I(\mathrm{device})}$$

where *I*(Potodiode) is the photocurrent, in which the dark current is subtracted, detected by the photodiode (Hamamatsu S1336-8BQ) placed just below the EL device; *I*(device) is the device current; *R* is the responsivity of the photodiode; and *g* is the configuration factor of our measurement setup, which we estimated was 4.

Brightness was also calculated from the EQE value and the EL spectrum. EL × CIE (Commission Internationale de I’Eclairage) gives the typical human response. Total luminance intensity was calculated by integrating the EL spectra over the whole wavelength range. Brightness was then calculated by dividing by the active device area and 2π, as shown in the equation below:

(4) $$\mathrm{Brightness}\;\mathrm{or}\;\mathrm{Luminance}\;\left(\mathrm{Cd}/m^{2}\right)\;=\frac{683.002\;\times \;\mathrm{EL}\;\mathrm{area}\;\mathrm{under}\;\mathrm{the}\;\mathrm{curve}\;\mathrm{normlaized}\;\times \;\mathrm{CIE}\;\times \;I\;\left(\mathrm{device}\right)\times \;EQE}{2\pi \times \mathrm{Device}\;\mathrm{Area}}$$

## 3. Results and Discussion

**Figure 1:**
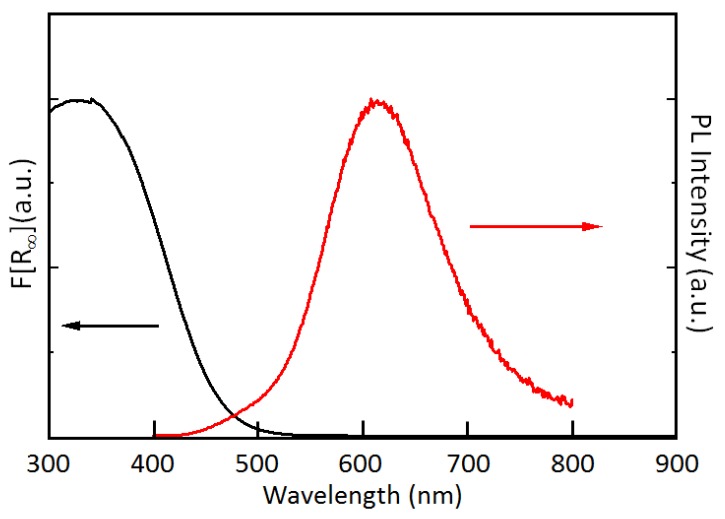
Photoluminescence (PL) and plots of F[R_∞_] vs. wavelength for the powder form of the decane-terminated silicon quantum dot (SiQD-De) specimen. F[R_∞_] of the powder form is the Kubelka−Munk function, with F[R_∞_] = (1 − R_∞_)^2^/2R_∞_.

The combined UV−VIS absorbance and scattering were studied via the Kubelka−Munk analysis, as shown in Figure 1. We prepared a SiQD-De on a film covering a 0.5 mm thick quartz glass substrate. The very broad spectrum with a peak at 340 nm might be due to some polydispersion of SiQD-De. The PL spectrum peak at 617 nm exhibits a narrow emission line but has a tail in a longer wavelength, possibly due to polydispersion. The SiQD-De has a diameter of 1.8 nm according to the Scherrer broadening analysis, corresponding to the PL band peak at 617 nm and 8% of an absolute PLQY. The decay time of the PL was approximately ~15 s due to the energy structure retaining the indirect bandgap character, corresponding to the literature [23].

**Figure 2:**
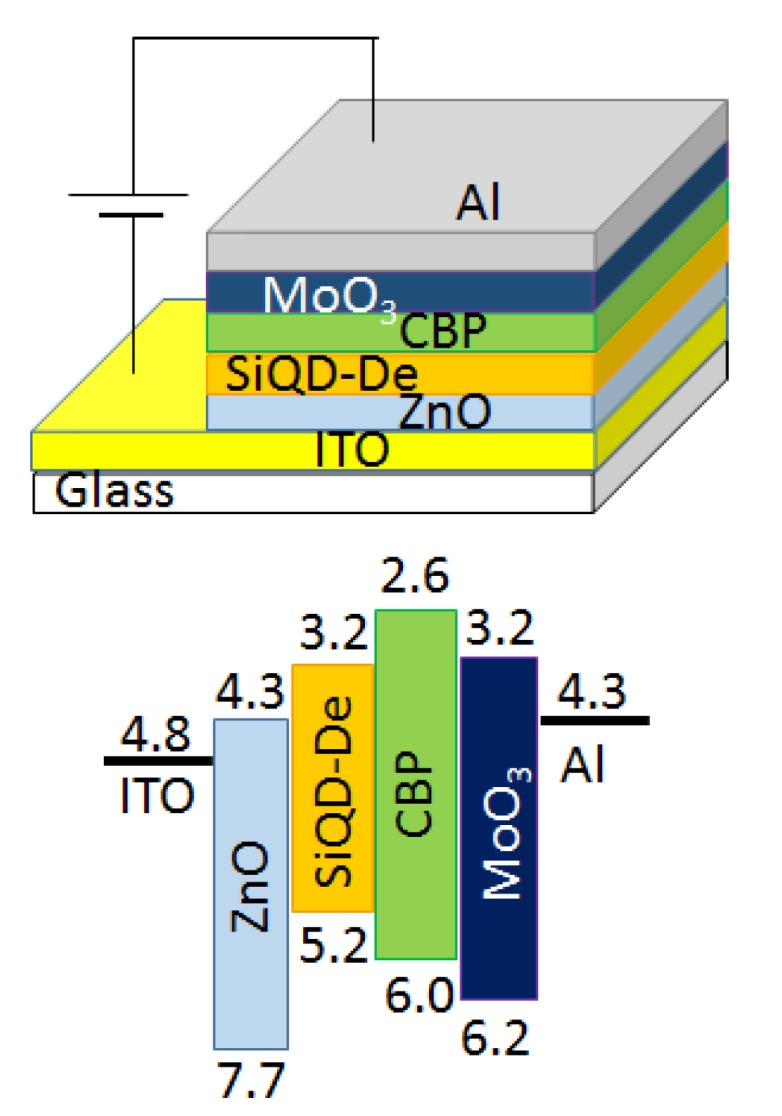
Schematic representation and flat energy band diagram of the pale-orange-light-emitting silicon quantum dot light emitting diode (Si-QLED) with an inverted device structure.

Figure 2 schematically illustrates a device architecture and its flat energy band diagram for our Si-QLED that exhibits the EL spectrum peaking at 620 nm. The device was constructed on a 150 nm thick layer of the ITO cathode (resistivity = 10−14 Ω/sq). The anode was a 150 nm thick Al film deposited under a vacuum. As shown in the drawing, the QLED consisted of an inverted device architecture with a multilayer structure, as reported in the literature [30,31]. In contrast to the conventional device structure, the electrons and holes were injected from the ITO and Al electrodes in the inverted device structure, respectively. The multilayered structure used here has constituent layers in the following order: ITO/ZnO/SiQD-De/CBP/MoO_3_/Al. The local surface work function of the MoO_3_ film was measured by photoelectron yield spectroscopy as 6.2 eV. According to the previous paper [30], the values of the work function of MoO_3_ films are influenced by the chemical composition of the films. In this work, a 30 nm thick MoO_3_ film deposited in 10^−5^ Pa vacuum conditions provided a local surface work function as low as ~6.2 eV. The electrons are transported to the SiQD layer through the electron injection and transportation layers (EIL/ETL) of the ZnO nanocrystal layer. On the other hand, the holes are transported to the SiQD layer through the hole injection and transportation layers (HIL/HTL) of the MoO_3_ and CBP layers. As illustrated in the energy diagram, the layer of ZnO exhibits a low electron affinity (−4.3 eV), being close to the value of the ITO, to facilitate the injection of electrons, leading to a low turn-on voltage. Furthermore, we expect that a low value in the work function of the ZnO layer (−7.7 eV) possibly suppresses a hole leakage current.

**Figure 3:**
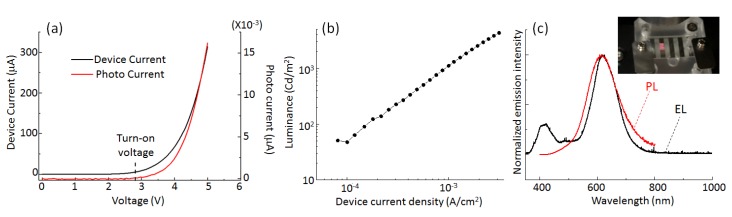
(**a**) Device I−V characteristics (black line) and photodiode I−V characteristics (red line), (**b**) luminance−current density characteristics, and (**c**) a typical electroluminescence (EL) spectrum at the operation voltage of 5 V (PL spectrum of the corresponding SiQD-De dispersed in chloroform). A photograph demonstrates a representative pale-orange-light-emitting quantum dot light emitting diode (QLED).

**Figure 4:**
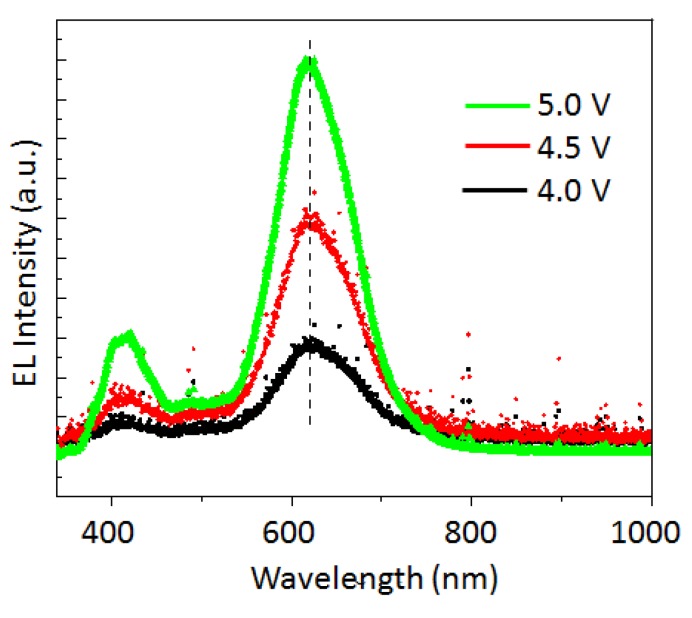
EL spectra at three different bias voltages.

**Figure 5:**
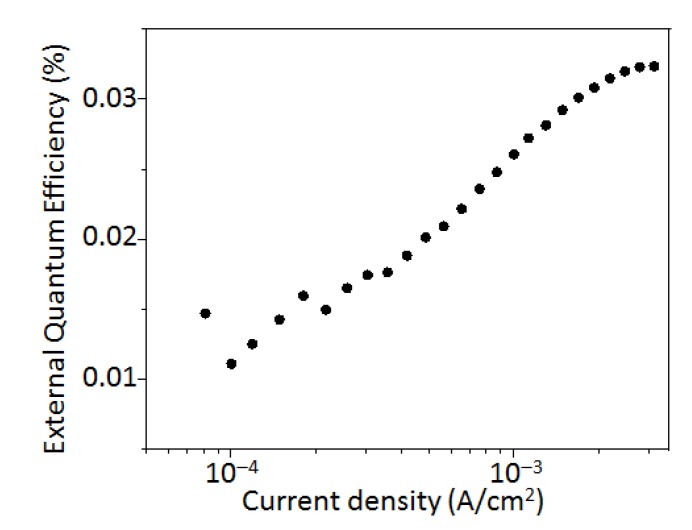
External quantum efficiency (EQE) versus device current density.

Figure 3 illustrates a typical performance of the Si-QLED in terms of the device current−voltage, the photocurrent−voltage, the luminance−voltage, and the EL spectrum compared to the PL spectrum. Figure 3a shows the current−voltage (I−V) characteristics along with the photodiode J−V characteristics. A calibrated Si photodetector (Hamamatsu S1336 8BQ, Hamamatsu Photonics) coupled with a Keithley 2423 was used for this measurement. The number of photons, collected directly with the photodetector, emitted from the ITO side, increased with the current. The turn-on voltage, which is defined as the minimum applied bias where the QLED starts to emit light, was estimated from the photodiode J−V characteristics. The estimated turn-on voltage was 2.8 V, which is as small as the value of the normal Si-QLED emitting the light peaking at 625 nm [27]. It is reported that the turn-on voltage is influenced by the device composition rather than the QD size [27]. Therefore, the observation of the low turn-on voltage for our device confirms the presence of a small barrier height for the charge injection into the photoactive layer from the electrodes. Figure 3b plots the typical luminance curves as a function of the applied voltage. The luminance reaches a value as high as 4200 cd/m^2^ at 5 V. The EL spectrum has a peak at 620 nm which shifts 3 nm to red when compared to the corresponding PL spectrum, as shown in Figure 3c. The EL spectrum is as narrow as 95 nm (~283 meV) fwhm, as evidenced in Figure 3c, and is slightly narrower than the PL spectrum. The observation of the negligible spectral shift (~3 nm) and the value of fwhm smaller than the PL linewidth indicates the effective suppression of the quantum-confined Stark effect, although further study is needed for clarification of the mechanism. The inset of Figure 3c shows a photograph of the QLED operating at 5 V, indicating that the light emitted from the QLED is sufficiently bright and vivid to be visible to the naked eye even in an illuminated room. However, the color purity of the pale orange was lower than our expectation in spite of the narrow linewidth of the spectrum without an emission tail. The low purity of color might be due to the appearance of another EL spectrum peaking at around 420 nm, while such a blue emission is not observed in the PL spectrum. The EL spectra exhibit the voltage dependence, as shown in Figure 4. The spectral profile at 4 V bias shows the EL peak at 620 nm, and we see a small luminance contribution (λ_em_ = ~420 nm) from a layer among the multilayers, leading to the low purity of the pale-orange color. This parasitic EL emission at 420 nm might originate from the neighboring compositional layer of ZnO. As the voltage increases, the parasitic EL intensity from the layer of ZnO increases. On the other hand, the EL intensity originating from the ncSi-De layers also grows under increasing applied bias voltage. There are two possible reasons to explain the appearance of the parasitic emission. First, according to the flat energy band diagram, there is an energy gap of 0.9 eV between the ZnO and the ncSi-De layers. Due to this energy barrier that blocks the carrier transportation, some of the electrons injected from the ITO electrode remained in the conduction minimum of ZnO, yielding the recombination for blue EL. The other possible reason is that this unwanted emission in the blue range appears due to an insufficient thickness of ncSi-De [28]. A further extension of voltage is needed to discuss the underlying physics in which the parasitic emission happens. Figure 4 demonstrates a couple of advantages of the inverted device structure. First, there is no shift of the EL peak with increasing operation voltage, indicating that the EL emission at 620 nm originates solely from the QD layer even in the high applied voltage range. In contrast, it is well known that the EL peak shift to blue for conventional structures of the QLED of semiconductors includes Si [25,26,32]. Second, the EL spectral shape is independent of driving voltage. It is important to note that emission spectral characteristics, such as EL peak position and shape, are not influenced by the presence of the parasitic emission spectrum. Therefore, the observed stability of the EL characteristics could be due to a good conductivity in the band alignment of HTL which leads to the difficulty in the buildup of the band-filling. In terms of the EL stability under increasing operation voltage, the use of metal oxide layers (i.e., ZnO and MoO_3_) for the EIL/ETL and the HTL/HIL takes advantage of the inherent robustness and protection of the interlayers from oxidation. We measured the photocurrent as the EL output with a photodetector to estimate the EQE and plotted the values in Figure 5 as a function of the injected current density. The peak value of EQE was estimated to be 0.033%, which is currently a record value for a Si-QLED operating in the pale-orange emission range. An enhancement of the EQE might be obtained by a good band alignment of our inverted device structure. Decreasing the degree of the charged QDs, which leads to carrier loss due to Auger recombination, would contribute to the enhanced EQE [33].

## 4. Conclusions

A SiQD-based QLED, exhibiting a narrow EL spectrum peaking at 620 nm, was produced via the solution-processed method. In this work, we synthesized a pale-orange luminescent sample of a SiQD with 8% PLQY and used it for an active layer of QLED. The QLED, consisting of multilayers of an inverted device structure, emits a pale-orange emission with 0.03% EQE, which is bright enough for confirming the emission by the naked eye even in an illuminated room. This work expands the tunable emission range that is currently limited to between 640 nm and 850 nm. The coefficient of optical absorption for the SiQD is significantly lower than those of QDs of II–VI and III–V semiconductors, because SiQDs retain the indirect bandgap structure, consistent with the experimental observation of a long PL decay time on a microsecond scale. Therefore, it is reasonable to consider that a SiQD is not a good phosphor adaption for phosphor-coated chips in the development of a liquid crystal display. However, a Si-QLED has the potential to become a good light emitter, because its poor optical absorption character does not influence the EQE. Further improvement of the optical performance of a Si-QLED toward the first commercialization of a heavy-metal-free QLED requires a dramatic enhancement of PLQYs even for visible-light-emitting SiQDs.
